# Supplementary material for: Are carbon emissions trading and green financial instruments synergistic? -Comprehensive quantitative research based on content analysis
Source: PLoS One. 2024 Mar 7;19(3):e0298601. doi: 10.1371/journal.pone.0298601 (PMC10919601; doi:10.1371/journal.pone.0298601)
Supplement: S1 Data — (ZIP) [file pone.0298601.s001.zip › Data/地级市Z_F工作报告.zip!/║┌┴·╜¡/┤≤╟∞╩╨/└┤╫╘╣1⁄2╓┌║┼í╛┬φ┐╦╩2╛▌═°í┐_2010.docx]

各位代表：

　　现在，我代表市人民政府，向大会作工作报告，请予审议，并请市政协委员和列席会议的同志提出意见。

　　一、2009年工作回顾

　　过去的一年，是经历重大挑战和严峻考验，经济社会发展取得突出成就的一年。面对国际金融危机、原油价格下跌带来的巨大压力和不利影响，坚持市委“创建百年油田、壮大接续产业、建设宜居城市、构建和谐大庆，全力推进大庆科学和谐跨越发展”总体思路和“保速度、促发展”的主基调，坚定不移保增长、保民生、保稳定，扎实推进60件大事实事，圆满完成了市八届人大四次会议确定的目标任务。全市实现地区生产总值2120亿元，增长12.1%，连续两年超过全省平均增速；完成固定资产投资753.5亿元，增长25.2%，其中地方投资增幅74.5%；新增各类企业1450户，企业总数达到1.35万户，其中“三上企业”1044户，主营业务收入超亿元企业144户；地方经济总量达到820亿元，增长32.7%；在原油价格折半的情况下，完成地方财政总收入145.5亿元，增长9.8%；城镇居民人均可支配收入18181元、增长8.1%，农民人均纯收入6593元、增长18.8%；万元GDP综合能耗下降4.9%。

　　——石油稳产、石化增效、地方工业大幅跨越，工业经济逆势增长。实现工业增加值1675亿元，增长11.4%。石油工业稳步发展。生产原油4000万吨、天然气30亿立方米，实现增加值1035亿元、增长2.6%，有力地支撑了全市经济。石化产业快速增长。120万吨乙烯改扩建等大项目顺利推进，10万吨苯乙烯等18个重点项目建成投产，规模以上石化企业扭亏增盈，实现利税186.6亿元，增加值288亿元、增长63.5%。地方工业规模扩张。实现增加值380亿元、增长60.1%，增速20年来最高。扎实推进“三百行动计划”，新建续建千万元以上项目157个，其中亿元以上34个，建成投产91个；新增规模以上地方工业企业70家，装备制造业、农产品加工业、建材业增加值分别增长68.8%、61.5%、78.9%；培育发展地方品牌，驰名商标、知名品牌达到55个。高新区提速升级，实现工业增加值168.2亿元、增长60.2%，占地方工业的44.3%，获准建设东北地区首个国家创新型科技园区。经开区加快成长，林源工业区建设扎实推进，各专业园区竞相发展，全市园区工业增加值占地方工业比重达到64%，工业基础更加牢固。

　　——粮食丰收、牧业提质、住房道路加快改善，新农村建设成效显著。实现农业增加值80亿元，增长13.5%。“四大基地”扎实推进。百亿斤粮食基地有新突破，新建玉米膜下滴灌50万亩、旱田喷灌53万亩，发展农机合作社37个，粮食产量比上年增加11.5亿斤、达到78.8亿斤，创历史最高水平；安全优质牧业基地稳步发展，规模化养殖小区达到190个、养殖大户7500个，肉、蛋、奶产量分别增长17.2%、7.8%、16.9%，畜牧业实现增加值40.3亿元、增长14.3%；无公害绿色食品基地不断扩大，面积发展到412万亩，产品销往北京、上海、广州等地，打入俄罗斯市场；区域观光农业基地初具规模，建设20个特色乡镇、50个特色村，基地面积达到6万亩，带动农民增收6亿元。生产生活条件显著改善。改造泥草房4.2万户、14.8万农民住上新居，新建农村道路500公里、饮水安全工程252处、户用沼气1万处、大中型沼气工程16处，整体绿化村屯455个，造林21.4万亩。农村自来水入户率和住房砖瓦化率分别达到67%、64%。公共服务水平明显提升。新建扩建14所寄宿制学校、6个乡镇综合文化站、125个农家书屋。提高新农合标准，筹款总额、参合率等五项指标全省最高。在全省率先构建“五位一体”农村综合救助体系，提高低保标准和救助标准，发放保障金4513万元、救助金2116万元，农村保障水平进一步提高。

　　——市场兴旺、产业升级、消费强势拉动，服务业快速发展。实现服务业增加值310亿元，增长16%。商贸业繁荣。建设新潮商业区等14个亿元以上项目，新建社区连锁超市15家，新增农资店、农家店300家。城乡消费持续旺盛，全年落户新车5.5万辆、增长55%，商品房销售面积310万平方米、增长28.7%，发放住房公积金贷款55.2亿元、增长135.8%，家电和汽车下乡产品销售额3.4亿元。全市社会消费品零售总额达到502.6亿元、增长30.2%，增幅全省第一。旅游业升温。引进建设北国之春梦幻城，建设完善连环湖、鹤鸣湖等景区17个，新增国家3A级景区3个。成功举办第二届湿地文化节，以缔结“连理同庆”城市旅游联盟为牵动，跨区域联合打造旅游品牌，扩大城市影响力。全年接待游客700万人次，实现旅游收入19.6亿元。服务外包业壮大。外包产业园二期、龙凤创业园建成投用，金桥信息等一批骨干企业实力增强，华拓数码进入全球“业务流程外包百强企业”行列，外包企业达到223家、主营业务收入52.3亿元。大庆成为中国服务外包示范城市。物流业提升。神舟石化物流园启动建设，市粮食综合批发市场全面竣工。石油物流园投入运营，入驻企业200多家。金融业突破。金融机构积极主动参与地方经济建设，贷款余额达到327亿元，比上年增加78亿元。上海石油交易所大庆石油交易中心投入使用，哈尔滨银行、克拉玛依城市商业银行、中意保险公司入驻我市，获批8家小额贷款公司，全市金融机构发展到44家，资产规模超过2000亿元。大庆成为中国最具特色金融生态示范城市。

　　——完善规划、打造精品、抓好十项整治，城市建设整体跃升。启动重点工程60项，完成投资210亿元。城市规划进一步完善。面向国内外公开招标，高水平编制10项重点规划。科学谋划长远，编制完成城市发展空间战略规划和总体城市设计；立足高标准开发建设“两城”，编制完成庆南庆北新城总体规划；结合重点区域开发，编制完成青龙山、富强等地区控详规划，为提升城市建设水平提供了重要保障。交通体系更加健全。全市人民企盼多年的萨尔图机场建成通航，打开了沟通全国、联接世界的空中通道；大广高速大庆段、铁路客运西站、公路客运枢纽等重大工程顺利推进，大齐高速大庆段、萨大路改造、一中立交桥等一批重点路桥项目如期投用，全市新建公路826公里，公路总里程达到2.4万公里。基础功能不断提升。东城水库主体工程竣工，主城区生活垃圾无害化处理厂开工建设，八百垧等3座生活污水处理厂和10座垃圾中转站投入使用，更新供水管网1.7万米、供热管网3万米，改造换热站23座。环境秩序明显优化。巩固国家卫生城创建成果，深入开展城市环境“十项整治”，整理41个小区环境，拆除违建40万平方米，规范集贸市场600多个，交通秩序顺畅，市容环境更加整洁。联通市区两级数字城管平台，视频监控覆盖重要街路、物业小区及公共场所。生态建设继续加强。黎明河带状公园建成投用，雨污分流、水清岸绿，成为怡人的休闲景观区。完成燕都湖和新潮湖治理，新建生态园6个，还建旧城区绿地33公顷，继续推进油田百里生态长廊工程，新建城市绿地1423公顷，植树170万株，建成区绿化覆盖率达到42.6%，人均公共绿地12.9平方米、增长20.6%。 ——理顺机制、扩大招商、对外贸易快速增长，改革开放扎实推进。重点领域改革进一步深化。理顺庆南、庆北新城管理体制，平稳推进驻外办事机构改革、市属粮食企业改制，继续完善国库集中支付制度，建立市内区域间经济合作机制。招商引资取得新突破。引进项目440个，其中高新技术和外资项目36个、亿元以上项目54个，实际到位资金153.7亿元、增长32.4%，创历年最好成绩。对外贸易逆势攀升。在外需低迷、全国全省利用外资下滑的形势下，完成进出口总额11亿美元、增长34%，实际利用外资1.4亿美元、增长19.7%，增幅均居全省前列。对上争取成效明显。171个项目得到国家和省里支持，争取资金30.3亿元。企业上市有新进展。瑞好集团、鑫隆公司分别在加拿大和新加坡成功上市，上市企业达到15家，境外上市企业户数居全省首位。

　　——整合资源、完善体系、增强公共服务能力，社会事业全面发展。教育事业有新提升。推进资源整合，撤并学校140所，新建扩建19所，优化了教育布局。93所义务教育学校通过标准化验收，新增省级示范高中3所，市区学生享受优质高中教育比例达到81.5%、全国领先，高考成绩继续保持全省第一。积极发展职业教育，中等职业学校招生增长39%，创近十年新高，建设中专晋升国家级重点职业学校。支持发展高等教育，高校办学水平、科研实力不断提升，在校大学生达到6.4万人。卫生事业有新变化。17家医院被评为国家级百姓放心医院，6家被评为国家级示范医院。社区卫生服务覆盖率达到96%。市二医院成功完成干细胞移植，填补省内空白。油田总医院住院三部、石化医院门诊楼主体工程竣工，市中医院新院投入使用。有效防控甲型流感，应对突发公共卫生事件能力进一步提高。人才和科技事业有新进步。出台人才优惠政策，设立人才发展专项资金，畅通流动渠道，健全培养使用机制，“六支人才队伍”不断壮大。加强科技创新体系建设，完善成果转化机制，市科技专利成果转化中心建成投用，大庆石油学院科技园成为国家级大学科技园。省科学院大庆分院、大连工业大学大庆生物技术研究院组建完成，推动实施产学研合作项目158个，新建科研成果推广示范基地35处，科技进步对经济增长贡献率达到48.7%。文体事业有新发展。加快提升文体功能，石油科技博物馆建成开馆，西城区体育馆、奥林匹克公园、城市规划展示馆等重点项目如期推进。培育发展文化产业，文化创意产业园启动建设，百湖艺术群落引入艺术家82人。《鹤鸣湖》荣获国家舞蹈最高奖项荷花奖。“书香大庆”等群众文体活动深入开展，第十届地企联合运动会成功举办，市民文明素质不断提升，大庆成为全国文明城市。

　　——加强社会保障、抓好民生实事、促进社会和谐稳定，人民生活进一步改善。坚持把改善民生作为“保速度、促发展”的出发点和落脚点，民生投入持续加大，公共服务优化提升，百姓关心关注的热点难点问题得到较好解决，人民群众的幸福感和满意度不断增强。社保体系进一步完善。积极推进“五险”扩面，净增21.5万人，解决了困扰多年的“五七工”、“家属工”养老参保问题；提高企业退休人员养老金标准，月人均增资116元；健全城镇“十位一体”低保综合救助体系，救助范围向临近低保的低收入群体扩展，发放保障金1.3亿元、救助金4051万元。平安大庆建设成效明显。加强群众工作和社会管理，实施“六调联动”，推进依法治市，实现重要敏感时期“五个确保”工作目标。深入开展信访调处专项行动，信访积案得到有效化解；组建城市应急指挥中心和12个专业应急救援中心，突发事件应对体系和处置能力进一步提升；加强社会治安综合治理，八类主要刑事案件下降14.9%、命案下降25.5%，十年来最低；深入开展安全生产“三项行动”和五个专项整治，各类安全事故下降12.4%。全市信访及公共安全工作整体水平全省领先。九件实事全面落实。改造萨中、龙凤南砖厂等棚户区37.1万平方米，建设安置房29万平方米，困难群众居住条件进一步改善；整理老区闲置公建和裸露地，建设地下车库、停车场、运动场、休闲广场16处、32万平方米，老居住区环境和功能有新提升；改扩建主城区街路30条、118.8公里，城区交通更加顺畅；完成红岗、大同等4万户居民燃气入户，城区管道天然气入户率达到80%；拓展就业渠道，实现城镇新就业5.4万人、城镇登记失业率控制在4.1%，新转移农村劳动力2万人，城乡就业稳定增长；通过福利彩票募集资金1.2亿元、高标准建成第二福利院，改造提升农村敬老院29个，私立老年公寓发展到35家，新增社区日间照料室63个，为90岁以上高龄老人发放生活补贴，大庆作为国家试点城市，社会化养老迈出新步伐；改善人才居住条件，开工建设1.7万平方米的人才公寓；新建改建16所乡镇卫生院，乡村医生培训率100%，农村医疗卫生水平有新的提高；查处食品药品违法案件1900多起，群众健康得到有效保障。

　　回顾过去一年全市经济社会发展，我们在挑战压力下开局，在矛盾困难中开拓，在团结拼搏中奋进，走过了极不平凡的历程。

　　这一年，隆重庆祝新中国成立60周年、大庆油田发现50周年、建市30周年，极大地激发了全市上下的创业热情，鼓舞了干部群众的斗志和士气，为推动大庆科学和谐跨越发展注入了强大动力。

　　这一年，保原油稳产、保投资强度、保重点企业、保项目建设、保消费规模、保财税增收，齐心协力“保速度、促发展”，在异常严峻的形势下，圆满完成了“保10争12”任务。

　　这一年，油化支撑主体，地方勇担重任，战线强力推进，县区各保一方，经济逆势增长，城建整体跃升，社会和谐稳定，财政收入、居民收入、投资消费、对外贸易等均创历史新高。

　　这一年，落实市委部署，坚持“负责任地干事、干负责任的事”，推进“行政提速、管理创新、执行彻底、结果问效”，在发展实践中考验锻炼了公务员队伍，改善优化了服务环境，有效提升了政府执行力。

　　各位代表，我市经济社会发展取得的可喜成就，得益于市委的坚强领导，得益于人大、政协及民主党派、社会各界的监督支持，得益于石油石化大企业和驻庆中省直单位的鼎力相助，得益于全市人民的团结奋斗。在此，我代表市政府，向全市人民，向所有为大庆经济社会发展做出贡献的同志们、朋友们，表示崇高的敬意和衷心的感谢！

　　同时，我们也清醒地看到，全市经济社会发展还存在一些矛盾和问题，政府工作也有许多差距和不足。主要是：产业结构不够合理，地方工业相对弱小，第三产业比重偏低，新兴产业亟待壮大；城乡建设管理有待提升，基础功能仍需完善；城乡居民收入、保障水平、生活条件还有差距；社会公共服务发展不够均衡，就医、就学、就业等民生问题需要进一步解决；政府有些部门和工作人员思想不够解放，服务质量、办事效率不高，作风还不够扎实。对此，我们一定要高度重视，认真加以解决。二、2010年工作安排

　　2010年是实施“十一五”规划的收尾之年，也是承接“十二五”规划、推动大庆科学和谐跨越发展的关键之年。我们必须审时度势、科学应对，努力把握发展的主动权。

　　当前，国际金融危机影响依然存在，不可预测因素仍然较多；国内经济企稳向好，但基础还不够牢固；石油价格反弹回升，仍有可能出现波动；各地新一轮扩张发展态势日趋明显，竞争压力越来越大。这些复杂情况，给我们带来的挑战和困难很多，保速度、促发展仍然是今后一段时期大庆发展的重要任务。

　　党中央、国务院和省委、省政府对大庆发展寄予厚望，胡锦涛总书记指示我们“弘扬大庆精神，推动科学发展”，温家宝总理要求我们“着力培育接续产业，加快大庆城市转型”，省委希望我们“用十年左右时间把大庆建成中国最美的城市之一”。市委提出到2016年提前四年全面建成小康社会，全市人民对过上更加美好的生活充满期待。面对这些期望重托，我们深感责任重大，在优化结构的基础上发展经济、在更高更好的标准下建设城市、按照均衡等质的方向改善民生，是党和人民赋予我们的光荣使命。

　　国家继续实行积极的财政政策和适度宽松的货币政策，出台进一步振兴东北地区等老工业基地若干意见，政策机遇十分宝贵。原油4000万吨稳产为发展提供了根本支撑，石化120万吨乙烯改扩建等大项目形成新的发展引擎，地方经济高速增长成为发展的生力军，城乡消费持续升温，投资规模逐年扩大，产业基础更加坚实，经济内生动力不断增强。特别是去年应对危机，市委提出“保10争12”，我们在逆势发展中积累了宝贵经验，坚定了信心决心。只要我们求真务实，埋头苦干，就一定能够实现更好更快发展。

　　在大庆加快发展的关键阶段，市委七届七次全会着重强调，要打牢产业基础、市政基础、人文基础、体制基础。这“四个基础”，是实现“五个跨越”的条件、加快“三个发展”的支撑、推动“两个转型”的根基、完成可持续发展的保障。这“四个基础”，既是当前任务，又是长远大计。打牢“四个基础”，要求我们必须抢抓历史机遇，珍惜来之不易的大好局面，保持强劲旺盛的发展势头，在加速发展经济、加快建设城市、繁荣社会事业、努力改善民生方面取得突破性进展。打牢“四个基础”，要求我们必须完成好“十一五”规划目标，科学编制“十二五”规划，稳定石油、壮大石化、扩张地方经济，千方百计引建龙头项目，强化服务培育骨干企业，集中力量打造知名品牌，保持经济12%左右增长速度，努力形成经济的持续高增长期；围绕建设生态自然现代宜居城市，拉开城市框架，创新建城理念，加快城建进程，扩大投资、深度融资、经营筹资，建设一批重大基础设施，努力形成城市的集中建设期；以加快教育、卫生、文体资源整合为主线，以建设大型功能项目为牵动，以完善公共服务体系为目标，瞄准先进地区抬高发展标准，统筹城乡提高等质化水平，努力形成社会事业的快速进步期；持续加大民生投入，集中办好惠民实事，大幅度提升城乡居民收入水平，大范围提升城乡社会保障能力，大力度提升群众的安全感、幸福感和归属感，努力形成人民生活水平的显著改善期。

　　2010年，政府工作总的要求是：以科学发展观为指导，深入贯彻市委七届七次全会精神，抢抓战略机遇，打牢四个基础，突出优化经济结构和转变发展方式，大力提升城市功能和改善生态环境，努力增强县域经济实力和缩小城乡差距，着力改善人民生活和维护社会和谐安定，加快构筑科学发展和谐发展跨越发展新优势。主要预期指标是：地区生产总值增长12%，固定资产投资增长35%，社会消费品零售总额增长30%，城镇居民人均可支配收入增长10%，农民人均纯收入增长17%，万元GDP综合能耗下降4.1%。

　　（一）始终坚持“四共”方针，尽心尽力服务石油石化大企业发展。突出石油石化重要地位，围绕“五大基地”建设，主动及时高效服务。产能服务要全面跟进。支持油田企业立足长垣、稳定外围、加快海塔开发，提高采收率，增加可采储量，确保4000万吨稳产。全面完成萨中等棚户区拆迁，还油还绿；对油田用地、打井拆迁等具体问题，特事特办、全力保障；保持高压态势，加强一线警力，严厉打击涉油违法犯罪活动。项目服务要全程支持。对120万吨乙烯改扩建项目，主动提供全方位服务；对30万吨聚丙烯二期、50万吨C4综合利用、5.2万吨聚丙烯酰胺等大项目，在规划、用地、拆迁等方面全力支持；对百万吨合成氨扩能改造项目，继续与大企业一道向上争取，把各环节工作落实到位，争取早日开工建设。生活服务要继续提升。完善大企业所在区域城市功能，加快推进西城体育馆、西宾跨线桥、油田热电厂贮灰池迁建等重点项目，开发铁路大庆西站地区、青龙山地区，建成现代都市的标志性新区。支持采油厂矿、化工厂区、职工生活区的基础设施、生态环境、服务功能建设，共建和谐矿区。 （二）强力推进“三百行动计划”，实现地方工业在扩张中优化。继续保持地方工业增长强势，增速达到55%以上。努力多上项目。新建续建重点项目160个，力促忠旺铝业、220吨阿维菌素、5万吨果蔬粉等109个新建项目早日开工，推动大丰抽油机、九环菊芋、博润玉米三期等一批项目如期投产。突出扶壮企业。支持企业上新项目、开发新产品，搞好扩能改造，新增规模以上企业100家；推动天泰生化、益朗司特等骨干企业提档升级、集团化发展，支持华科建设全国最大C5、C9树脂加工基地，力争主营业务收入超亿元的企业达到100家；争取肇融集团等3家企业上市。大力培育品牌。实行重点品牌培育专项奖励，抓好本地优质特色产品推介和品牌申报，支持大庆牌汽柴油等28个产品争创省级以上名优商标和品牌，扩大大庆制造、大庆创造的市场影响力。加速“三区”扩张。高新区要建好主体产业区、提升兴化园区、扩大宏伟园区，在引进战略龙头企业上有大作为，实现工业增加值两年倍增目标。经开区要加速项目集聚，完成后四厂搬迁投产，抓好华能电厂等大项目，规模以上企业达到50家。林源工业区要完善功能区规划，全面启动化工和生物产业园，引进建设大项目，确保10万吨改性沥青等项目投产达效。强化科技支撑。支持省科学院大庆分院等科研院所增强研发实力，引进建设北大荒集团大庆生物工程技术研究院，组建生物、装备制造、服务外包三个技术创新联盟，探索建立科技投资创业引导基金，发挥好科技专利成果转化中心等平台作用，切实增强科技对经济的支撑能力。

　　着力转变发展方式，优化经济结构，培育壮大战略性新兴产业，实现增加值超百亿元。加快发展新材料产业，支持汉维长垣、路通科技、联塑集团等企业扩大产能，重点发展精细化工材料、合成材料、复合材料和电子材料。加快发展新能源产业，推进瑞好、天润等风电项目，搞好地热综合利用和深度开发，提升风能、地热、太阳能产业化水平。加快发展新型环保产业，大力发展环保设备、环保服务、环保产品，启动建设大庆生态卫星城、喇嘛甸低碳工业园，积极争取国家低碳经济试点。加快发展生物产业，围绕拓展延伸生物农业、生物医药、生物能源、生物制造和生物环保等产业链条，建好生物产业园区，培育壮大展华、巍天等骨干企业，打造省级生物产业基地。加快发展信息产业，重点发展电子元件、工业软件和行业应用信息产品，支持同创、三维、佳昌等企业做大做强，扩张产业规模。加快发展现代装备制造业，立足油化产业优势，瞄准高端领域，在先进采油装备、大口径输油输气设备、精密管阀等方面取得突破，支持力神泵业、大丰集团等企业开发高端产品。

　　（三）发展壮大县域经济，整体提升新农村建设水平。坚持以城带乡、以工促农，加快产业化壮大县域经济，推进基地化发展现代农业，搞好城镇化建设现代农村，确保粮食持续增产、农业持续增效、农民持续增收。推进四大基地建设。围绕提升市场化、水利化、机械化、标准化、规模化水平，推进粮食基地建设，新建玉米膜下滴灌106万亩、旱田喷灌62万亩，组建农机合作社46个，高产田发展到650万亩，粮食总产量力争达到90亿斤。推进牧业基地建设，继续实施“六吨奶”工程，重点发展养殖小区和养殖大户，抓好重大疫病防控，畜牧业增加值增长10%以上。推进绿色食品基地建设，突出大豆、水稻等优势农产品，整县推进无公害产品认证、产地认定，全市基地面积发展到450万亩。推进观光农业基地建设，提升特色观光乡镇和观光村水平，设施农业规模达到10万亩以上，配套完善休闲娱乐功能，提高综合效益。壮大县域经济实力。打造产业特色鲜明、园区竞相发展、乡镇快速崛起的县域经济发展格局。培育壮大粮豆薯、肉蛋奶、果菌菜等产业链条，推进玉米、皮革、轻纺等专业园区扩张升级，带动大基地，促进产业化。在加快发展城关镇的同时，集中建设10个设施完备、产业成形、要素集聚、特色鲜明的“经济重镇”，继续建好百个示范新村，提升城镇化水平，带动县域经济快速发展。加强基础设施建设。新建农村公路320公里、饮水安全工程256处、大中型沼气工程50处；深入实施农村环境整治工程，植树造林20万亩，绿化村屯200个，整治村容村貌，促进农村环境明显改善。不断深化农村改革。完善农村土地经营制度改革，扩大土地规模经营；推进农村金融改革，抓好农村资金互助社试点；完成集体林权制度改革，形成良性发展机制。积极发展产业协会、龙头企业、专业合作社、基地农户“四位一体”的新型农合组织，提升农业的组织化、市场化程度，发挥对现代农业的促进作用。

　　（四）着眼打造“六个名区”，促进服务业大发展大繁荣。把服务业作为调整经济结构、扩大内需、促进就业的战略性支柱产业，着力培育区域品牌，服务业增加值增长20%以上。组合开发，打造高档商贸核心区。突出重点区域，整体开发建设，构建新玛特、东安、龙南和五湖地区现代商贸圈。引进名企名店，建好万达中心商务区、西城汽车销售园、银亿商业区。扩大城乡连锁经营，新增社区店30家、农家店200家。整合做活，打造区域物流中心区。整合区域市场，做大石油物流园；发挥石化产品产地优势，建好神舟石化物流园；利用地理区位条件，提升港桥物流园；依托萨尔图机场，辟建空港物流园。积极培育壮大物流企业群体，发展园区生产物流和都市配送物流，加快现代物流体系建设。深度开发，打造特色旅游精品区。推进北国之春梦幻城、龙凤湿地公园等重点项目，提升连环湖温泉城、林甸温泉养生基地、大同生态农业旅游区，谋划建设大庆工业遗址公园，培育建设旅游名点、名镇、名区。高水平办好第三届湿地文化节，扩大肇源莲花节、杜尔伯特那达慕、大同采摘节等特色节庆影响力。发展旅游中介，做大旅游市场，旅游收入增长35%以上。放开放活，打造中介金融聚集区。加快金融产业园建设，发展证券、保险、担保和风险投资企业；引进浦发银行、兴业银行，发展小额贷款公司；探索企业债券融资，鼓励金融产品和信贷模式创新；搞好银企对接，增强金融对经济的支撑作用。提升壮大，打造服务外包优势区。建好外包产业园三期、黑龙江软件园、石油工程技术服务园，培育壮大华拓数码等骨干企业群，外包企业发展到300家。塑造品牌，打造文化创意示范区。加快建设文化创意产业园，引进建设“新华08”石油资讯国际总部、黑龙江新媒体基地，重点发展动漫游戏、数码设计、影视制作、文化会展、资讯传播等业态，促进文化产业加快升级。

　　（五）突出抓好重点工程，加快建设生态自然现代宜居城市。巩固“三城联创”成果，坚持功能补位、老区提升、新区拓展，突出新城整体推进、项目集中开发、环境纵深治理、生态深度优化、管理全面升级，进一步打牢市政基础。坚持高点站位，完善提升规划。突出先进理念，塑造风格特色，做深西城区总体规划，做细城市重点建设区域控详规划，做优道路、绿地、供热、给排水等专项规划，做好滨水环境建设规划，做精地标性、景观性建筑单体设计。实质推进“两城”，拉开城市框架。立足适度超前、满足需要，加快庆南、庆北新城水电气讯路等基础设施建设。着眼增强产业驱动力，加快林源工业区、临空产业区载体建设。围绕完善教育、医疗、商贸等功能，加快大同三十五中学、客运站迁建，红岗重点高中、商贸城等项目建设。实施集中突破，建好重大项目。高标准建设60项重点工程。抓好世纪大道整理及龙凤湿地立体跨越、大广高速大庆段、铁路大庆东西两站、公路客运枢纽等项目，配合建设哈大齐客运专线，提升交通功能；建好奥林匹克公园、新体育场及体校新校区、中央党校大庆教学基地、规划展示馆、市图书馆等项目，提升文体功能；启动龙凤南砖厂、中五路等地区建设，提升区域整体功能；完成房地产开发面积500万平方米，加大调控力度，实现良性健康发展。继续抓好“十项整治”，推进纵深管理。创新完善城市管理体制，强化属地责任，探索实行联合执法，落实“门前五包”责任制，积极构建大城管格局。扎实搞好萨大路市场整治，持续加大公共场所、城乡结合部、油田过渡带、背街小巷等环境治理力度，推动各项整治向四县延伸。完善数字城管系统功能，加快建设县级管理平台，进一步提高城市管理精细化水平。突出治水绿化，大规模开展生态建设。继续实施黎明河治理二期工程，搞好兰德湖、青龙湖等湖泊综合治理。继续推进油田百里生态长廊、西北风口防护林、松嫩两江堤岸防护林建设，突出抓好中三路、西干线、外环路等沿路绿化，重点建设东湖生态林、龙凤森林公园二期、五湖商务区生态林等7个绿化区块。动员全社会力量，开展沿街植绿、厂前覆绿、门前插绿、小区造绿。新建城市绿地1500公顷，植树210万株，建成区绿化覆盖率达到45%。对照国家新标准，严格整改、完善提升，确保顺利通过国家环保模范城复检。 （六）围绕实施“十大民生工程”，努力促进社会和谐。健全公共服务体系，抓好社会矛盾化解，探索社会管理创新，办好民生具体实事，打造人心和善、家庭和睦、人际和顺、社会和谐、生态和美的“五和”生活愿景。建设均衡公平的教育体系。优化资源配置，撤并学校56所，新建三永湖、富强地区等4所九年一贯制学校、8所农村寄宿制中小学校，城区义务教育学校标准化率提高30%，市区学生享受优质高中教育比例达到85%以上。统筹全市职教资源，筹建技师学院，创立公共实习实训基地，打造品牌专业，支持大庆职业教育学院建设国家示范高职院校，提高职业教育规模和水平。支持发展高等教育，培养优秀复合人才，建好大学科技园，促进高校科技成果转化，发挥高校在经济社会发展中的重要作用。建设优质便民的医疗体系。建立市带县区、县区带乡镇的医院管理机制，完善三级医院领办社区、乡镇医院领办村卫生所模式。建成投用油田总医院住院三部，启动建设市妇女儿童医院，支持综合医院与国内名院名校联合协作。健全公共卫生应急处置机制，提高重大传染疾病防控能力。建设功能完善的文化体系。以石油文化为根基，以都市文化为引领，推动文化事业繁荣发展。打造艺术精品，开发专业市场，有效利用文体设施。完善社区文体活动场所、乡镇综合文化站和农家书屋。继续搞好“书香大庆”、“激情之夏”等品牌载体活动，丰富群众文化生活。建设提升乒乓球、游泳、速滑等培训基地，深入开展全民健身运动。建设覆盖城乡的社保体系。继续推进“五险”扩面，吸纳农民工、非公企业员工、个体从业人员等参保。搞好城镇“十位一体”、农村“五位一体”低保综合救助，建立城镇临近低保低收入群体救助机制。提高新农合筹资和大病救助标准，让农民得到更好保障。抓好肇源县国家级新农保试点。建设和谐有序的社会管理体系。以“尊民意、聚民力、解民难、维民权、促民和”为主线，扎实做好社会管理和群众工作。健全完善“六调联动”机制，构建市、县区、乡镇街道、村屯社区“四级”群众工作网络，有效预防并及时化解社会矛盾。加强社区建设，加大社区投入，提高社区工作人员工资标准，搞好居委会换届选举，建好社区服务站，着力打牢基层基础，发挥社区在社会管理中的基石和桥梁作用。突出抓好信访工作，强化群众来访接待调处中心作用，推行信访“三级终结”，继续化解信访积案，积极做好矛盾排查调处，落实重大事项社会稳定风险评估机制，努力从源头减少信访问题。建设保障有力的公共安全体系。坚持“两保四大”工作格局，建立健全预防预警、快速反应、指挥调度、应急处置机制。加大社会治安打防控力度，加强城市报警和护卫系统建设，严厉打击各类违法犯罪。建立安全隐患群众有奖举报制度，以油气管线、危险化学品为重点，抓好企业生产安全；以整顿行车停车秩序、完善道路交通管理设施为重点，抓好交通安全；以建筑市场秩序、工程质量、施工现场为重点，抓好建筑安全；以食品药品、金融秩序、农业生产资料为重点，抓好专项整治，加强市场监管，努力建设平安大庆。

　　在加强社会建设的同时，着眼群众最急需、最直接、最现实的利益问题，办好与群众生活密切相关的十件具体实事。一是促进就业，稳定转移农村劳动力2万人，开发就业岗位5.5万个，城镇登记失业率控制在4.5%以内。二是城市棚户区改造，加快萨中地区等32处、61.4万平方米拆迁改造，其中市区11处、29.8万平方米，建设安置房23.9万平方米，基本完成市区棚户区改造任务。三是农村泥草房改造，改造完成5万户，惠及农民17.5万人，累计完成省下达全部任务的80%。四是垃圾污水处理，新建垃圾中转站10座，建成投用主城区生活垃圾无害化处理厂，建设四县污水处理厂和垃圾处理场。五是热源和热网改造，新增供热能力400万平方米，集中改造热网1万米，进一步加大供热行业监管力度，提高供热质量。六是供水系统改造，三年内完成新水源地、新水厂建设，整体改造供水管网，确保城市饮水安全。今年，完成东城水库建设，开工建设东城水厂，改造供水管线1.5万米，新建改建升压站2座。七是道路安全管理设施建设，建成道路交通控制中心，建设6个分控中心，新增12个灯岗、50套电子警察、2000个安全标志牌等设施，提高道路安全管理水平。八是建设特殊教育发展中心，为残疾人提供教育、康复、托养、职业培训、心理诊疗等综合服务。九是校园安全，推进校舍安全工程，加大校园治安、消防和交通安全治理力度，规范整治校园周边网吧、音像、图书等经营秩序，为青少年营造更好的学习成长环境。十是社会化养老，新增民办养老院10家，新建社区老年人日间照料室40个，推行居家养老服务，抓好国家、省、市三级社会化养老试点。

　　（七）激发释放“四个活力”，深化改革扩大开放。抓好重点领域和关键环节改革，积极拓展对外开放的广度和深度，努力构建有利于更好更快发展的体制环境。发展非公经济，释放创业活力。鼓励全民创业，降低准入门槛，拓宽创业领域，实行国家法律法规“非禁即入”，支持企事业单位科技和管理人员创办领办企业。加强创业服务中心和孵化基地建设，帮助企业争取资金、项目支持。开展创业培训，完善担保体系，优化创业和发展环境，力争非公经济增长30%以上。推进重点改革，释放体制活力。按照国家行政管理体制改革要求，抓好政府机构改革。分类实施事业单位改革，稳步推进医药卫生体制改革，启动文化管理体制改革，完成驻外办事机构改革，深化公务用车制度改革。抓好招商引资，释放经济活力。完善招商政策，力争引进吉利沃尔沃汽车、新恒基LED等重大龙头项目。创新招商模式，改革招商评价体系，确保招商实效，全年招商到位资金120亿元以上。扩大外经外贸，释放开放活力。加快石油物流园内陆港功能建设，申报争取航空口岸，扶持壮大外贸骨干企业，推动对外贸易升级。力争有进出口业绩的企业突破90家，进出口贸易总额增长20%以上。

　　（八）强化执行落实能力，全面提升政府行政效能。按照市委统一要求，深入开展“执政为民教育年”活动，始终坚持负责任地干事、干负责任的事，继续推进行政提速、管理创新、执行彻底、结果问效，着重突出“创新创造创优、大干实干快干”，高标准高质量完成各项目标任务。保持良好的状态开拓进取。坚定不畏艰难、敢于胜利的信心决心，秉持抢抓机遇、化危为机的思维理念，保持争分夺秒、百日攻坚的“机场速度”，形成比学赶超、争创一流的生动局面。采取管用的机制推进落实。坚持“三维分工”体制、“五个一”模式、“五定五保”措施，完善政府工作定期通报制度，抓好“三位一体”督办督查。探索扩大并联审批范围，推行现场办公制度化、常态化，加强协调配合，实行工作联动，坚决消除中梗阻现象。拿出创新的举措破解难题。把思维的起点放在干事成事上，把攻坚的重点放在打破瓶颈上，把工作的着力点放在创新突破上。对于能够牵动一个产业的立市大项目，就要敢于给大政策、大支持；对于影响项目建设的关键环节，就要打破常规，大胆突破；对于长期以来积累的矛盾问题，就要找准症结，敢下决心，从根本上理顺和解决。依靠务实的作风树立形象。切实精简文件、减少会议，推行政府绩效管理和行政问责。完善提升行政服务中心功能，深化政务公开。加强财政预算管理和监督，严格专项资金使用管理。认真落实党风廉政建设责任制，加强执法监察、廉政监察和效能监察，树立为民务实清廉的政府形象。

　　始终坚持市委统一领导，主动接受人大法律监督和工作监督，自觉接受政协和民主党派的民主监督。认真贯彻落实市委决策，认真执行市人大及其常委会决议，认真办理人大代表建议和政协委员提案，支持工会、共青团、妇联等群团组织开展工作。坚持科学民主决策机制，重大问题集体讨论决定。健全社情民意反映机制和重大事项专家咨询、社会公示制度。推进依法行政，规范行政许可行为，努力提高政府的法制化管理水平。

　　各位代表，做好今年的政府工作，任务艰巨而繁重，使命重大而光荣。我们要在市委的正确领导下，深入落实科学发展观，传承弘扬大庆精神、铁人精神，解放思想，与时俱进，居安思危，团结拼搏，为加快构筑大庆科学和谐跨越发展新优势而努力奋斗！
